# Comparative Validation of Conventional and RNA-Seq Data-Derived Reference Genes for qPCR Expression Studies of *Colletotrichum kahawae *

**DOI:** 10.1371/journal.pone.0150651

**Published:** 2016-03-07

**Authors:** Ana Vieira, Ana Cabral, Joana Fino, Helena G. Azinheira, Andreia Loureiro, Pedro Talhinhas, Ana Sofia Pires, Vitor Varzea, Pilar Moncada, Helena Oliveira, Maria do Céu Silva, Octávio S. Paulo, Dora Batista

**Affiliations:** 1 CIFC—Centro de Investigação das Ferrugens do Cafeeiro, Instituto Superior de Agronomia, Universidade de Lisboa, Oeiras, Portugal; 2 Computational Biology and Population Genomics group, cE3c - Centre for Ecology, Evolution and Environmental Changes, Faculdade de Ciências, Universidade de Lisboa, Lisboa, Portugal; 3 LEAF-Linking Landscape, Environment, Agriculture and Food, Instituto Superior de Agronomia, Universidade de Lisboa, Lisboa, Portugal; 4 Plant Cell Biology, Instituto de Tecnologia Química e Biológica António Xavier, Universidade Nova de Lisboa, Oeiras, Portugal; 5 Cenicafe, Manizales, Colombia; USDA-ARS, UNITED STATES

## Abstract

*Colletotrichum kahawae* is an emergent fungal pathogen causing severe epidemics of Coffee Berry Disease on Arabica coffee crops in Africa. Currently, the molecular mechanisms underlying the *Coffea arabica—C*. *kahawae* interaction are still poorly understood, as well as the differences in pathogen aggressiveness, which makes the development of functional studies for this pathosystem a crucial step. Quantitative real time PCR (qPCR) has been one of the most promising approaches to perform gene expression analyses. However, proper data normalization with suitable reference genes is an absolute requirement. In this study, a set of 8 candidate reference genes were selected based on two different approaches (literature and Illumina RNA-seq datasets) to assess the best normalization factor for qPCR expression analysis of *C*. *kahawae* samples. The gene expression stability of candidate reference genes was evaluated for four isolates of *C*. *kahawae* bearing different aggressiveness patterns (Ang29, Ang67, Zim12 and Que2), at different stages of fungal development and key time points of the plant-fungus interaction process. Gene expression stability was assessed using the pairwise method incorporated in geNorm and the model-based method used by NormFinder software. For *C*. *arabica—C*. *kahawae* interaction samples, the best normalization factor included the combination of *PP1*, *Act* and *ck34620* genes, while for *C*. *kahawae* samples the combination of *PP1*, *Act* and *ck20430* revealed to be the most appropriate choice. These results suggest that RNA-seq analyses can provide alternative sources of reference genes in addition to classical reference genes. The analysis of expression profiles of bifunctional catalase-peroxidase (*cat2*) and trihydroxynaphthalene reductase (*thr1*) genes further enabled the validation of the selected reference genes. This study provides, for the first time, the tools required to conduct accurate qPCR studies in *C*. *kahawae* considering its aggressiveness pattern, developmental stage and host interaction.

## Introduction

*Colletotrichum kahawae* J.M. Waller & P.D. Bridge, the causal agent of Coffee Berry Disease (CBD), is a highly aggressive and specialized fungal pathogen of coffee. This pathogen currently occurs in nearly all African regions where Arabica coffee (*Coffea Arabica* L.) is grown, particularly at high altitudes, ravaging plantations and causing up to 80% yield losses, if no control measures are applied [1–3]. The potential introduction of this quarantine pathogen into other continents represent a major concern and threat, particularly to high altitude Arabica coffee plantations that can also be found in Latin America and Asia. *C*. *kahawae* infects several coffee organs but maximum production losses occur when infection takes place in expanding green berries, leading to their premature dropping and mummification [2–4].

Functional and molecular studies to better understand *C*. *arabica—C*. *kahawae* interactions are of key importance to aid disease resistance breeding efforts. Real time quantitative PCR (qPCR) is currently the most accurate method for analyzing gene differential expression given its capability of detecting low abundance mRNAs and slight differences in the expression level. When compared to other methods used to evaluate transcript accumulation, such as Northern blotting, RNase protection assay, *in situ hybridisation*, and cDNA microarray technology, the main advantages of qPCR are its high sensitivity and specificity. However, in order to obtain reliable and reproducible results, proper data normalization is necessary [5].

Different normalization strategies have been proposed [6,7], but normalization of gene expression levels by reference genes (RGs) is most certainly the "gold standard", though the success of this procedure relies on the appropriate choice of RGs [8,9]. Typically, RGs consist of a group of constitutively expressed genes which are considered to be essential to maintain basic cellular functions, and are ubiquitously expressed in all cells of an organism, irrespective of tissue type, developmental stage, cell cycle state, or external signals [10]. However, some evidence shows that almost all genes seem to be regulated at some point under certain conditions and there are always some variations in transcript levels, so that none of the commonly exploited genes can be viewed as universal RGs [8,9]. So far, most studies have focused on validating a subset of commonly used RGs for a specific context. Although this seems to be a good strategy, such studies try to identify the best candidates from a small and *"a priori"* set of RGs, assuming that at least one or a few of them are suitable for the experimental context under study [11].

In order to avoid this potential bias, researchers have conducted genome-wide surveys to search for new RGs using different types of datasets such as microarray [11], EST libraries [12,13], tag-based approach serial analysis of gene expression (SAGE) [14] and more recently RNA-seq [10,15,16]. RNA-seq seems to be a powerful tool for identifying reference genes across a global transcriptome, providing a significantly more accurate measurement of transcript abundance [10].

Recently, a high-throughput RNA sequencing (RNA-seq) approach was applied to study compatible and incompatible *Coffea* spp.*-C*. *kahawae* interactions providing unprecedented information on the coding-genes putatively involved, and on their expression for both the host and the pathogen [17]. RGs suitable for gene expression studies in both resistant and susceptible coffee genotypes to *C*. *kahawae* were already established [18], but no normalization system was developed to study gene expression in the pathogen. Moreover, since the validation of RNA-seq data is ideally achieved through qPCR analysis, the establishment of a set of RGs for the pathogen is a crucial step to validate the fungal RNA-seq results.

In this study, we selected a set of 8 candidate RGs, including RGs previously used for other phytopathogenic fungi and a new set of genes retrieved from the analysis of several RNA-seq datasets of a compatible and incompatible *Coffea spp*.—*C*. *kahawae* interaction [17]. The stability of the candidate RGs was evaluated using four *C*. *kahawae* isolates bearing different aggressiveness patterns in a range of *C*. *kahawae* samples and *C*. *arabica*—*C*. *kahawae* samples. Furthermore, since *C*. *kahawae* is a hemibiotrophic pathogen with substantial biomass variation during the infection stages within the plant [19], an additional methodology for normalization of the *C*. *kahawae* biomass in *C*. *arabica*—*C*. *kahawae* samples was required. For that, the biomass quantification was carried out by measuring the fungal DNA by qPCR in the same *C*. *arabica*—*C*. *kahawae* samples used and accounted for in the expression analyses.

The best combination of RGs determined for each dataset was further validated by assessing the expression of two genes described as putatively involved in the early steps of the infection process of pathogenic fungi, namely a bifunctional catalase-peroxidase (*cat2*) and a trihydroxynaphthalene reductase (*thr1*) [20–26]. *cat2* was described as being involved in reactive oxygen species (ROS) detoxification by removing intracellular hydrogen peroxide (H_2_O_2_) and converting it into water and dioxygen [20–22, 26]. This protein has a crucial role in the first stages of the infection process, since previous studies revealed its involvement on pathogen protection against the generation of ROS by the host, one of the most rapid and dramatic defense reactions activated by the plant following the pathogen attack [20]. The trihydroxynaphthalene reductase protein encodes the *thr1* gene and was previously described as being involved in the first reduction step of the melanin biosynthesis pathway [23–26]. Melanin is important for pathogenicity in some plant pathogenic fungi since appressorial melanization is essential for penetration of host tissues [23–26].

The expression profiles evaluation of those genes with a different set of candidate RGs clearly demonstrates the impact of different normalization approaches in the final results. The global strategy applied on this work allowed, for the first time, the establishment of a new set of RGs suitable for gene expression studies in *C*. *kahawae*, considering its aggressiveness pattern or stages of fungal development and infection.

## Material and Methods

### Fungal isolates

Four *C*. *kahawae* isolates (CIFC/ISA/Universidade de Lisboa collection) bearing different aggressiveness patterns, as observed in CIFC´s routine screening tests, were used (Table 1): Ang 29 and Zim 12—highly aggressive isolates (leading to tissue complete necrosis in 6–8 dpi); Que 2- moderately aggressive isolate (leading to tissue complete necrosis in 10 dpi); and Ang 67—low aggressive isolate (leading to tissue complete necrosis in 20 dpi). These isolates were grown in 90 mm polystyrene Petri dishes containing malt extract agar (MEA, Oxoid, England) for 7 days under a photoperiod of 12 h at 22°C, in order to obtain conidia.

**Table 1:** Details on *Colletotrichum kahawae* isolates regarding its geographical origin and aggressiveness pattern in *Coffea arabica* (var. Caturra).

Isolate	Origin	Aggressiveness pattern
**Ang29**	Angola	High
**Ang67**	Angola	Low
**Que2**	Kenya	Medium
**Zim12**	Zimbabwe	High

### Inoculation of coffee hypocotyls

Coffee hypocotyls were used as a model material in controlled conditions, since previous studies have shown a correlation between the pre-selection test on hypocotyls and mature plant resistance to CBD in the field (r = 0.73–0.80) [27]. Seeds of *Coffea arabica* (var. Caturra; susceptible to CBD) were sown in seedbeds in a growth chamber (FITOCLIMA Walk-in 10000 EHHF, Aralab, Portugal) under controlled conditions (24–26°C, with 12 h photoperiod at 800 μmol.m^-2^.s^-1^ light and 75–85%relative humidity) during 7–8 weeks. Plantlets were collected after emergence (prior to cotyledon expansion) and hypocotyls were then inoculated and maintained as described by Figueiredo *et al*. [18]. Briefly, hypocotyls were sprayed with a conidial suspension of *C*. *kahawae* (3×10^6^ conidia.ml^-1^) and maintained in a moist chamber at 22°C in the dark for 24 h, and then under a 12 h photoperiod during the infection time-course.

### Sample preparation and collection

In this work, two types of samples were collected: *C*. *kahawae* samples, representing different stages of fungal development [ungerminated conidia, germinated conidia with mature appressoria formed after 18-22h on polystyrene or on leaves of *C*. *arabica* (hereafter referred as appressoria), and saprophytic mycelium] and *C*. *arabica—C*. *kahawae* samples, representing key steps of the infection process (melanized appressoria, beginning of penetration and early stages of necrotrophy).

#### *C. kahawae* samples

Ungerminated conidia were harvested from 7-to 10-day-old cultures, grown on MEA medium under a photoperiod of 12 h, into sterile water and cleared from mycelium using a borosilicate glass filter crucible with porosity 1. To produce saprophytic mycelium, 5 ml of conidial suspension (2 × 10^6^ conidia.ml^–1^) were inoculated in 25 ml of Potato Dextrose Broth (BD-Difco, USA) and grown for 3 days at 22°C. The mycelium was filtered off from the culture broth through a fine cloth mesh and washed with distilled water. The mycelium was lyophilized and stored at -80°C until use. *In vitro* appressoria were obtained following the methodology of Kleemann *et al*. [28] with some modifications. A monolayer of 15 ml conidial suspension (1 × 10^6^ conidia ml^–1^) was used and filter paper was applied to the liquid surface instead of the nylon mesh. The appressoria formed on polystyrene plates after 18–22 h were scrapped with 10 ml of sterile water containing 0.02% Tween 20 (Fisher Scientific, USA). The suspension was centrifuged at 5000 g for 10 min, and the appressoria pellet was collected, lyophilized and stored at -80°C. To obtain appressoria *in planta*, the abaxial surface of 12 young leaves of *C*. *arabica* (var. caturra) were sprayed with a conidial suspension (2 × 10^6^ conidia.ml^–1^) of each isolate, using an atomizer. The inoculated leaves were maintained in a humidity box for 24h at 22°C to allow conidia germination and appressoria formation. After this period, the leaves were air dried and a thin layer of nail polish was applied [29]. The nail polish, containing the fungal structures (as verified by microscopic examination of cotton blue-stained nail polish fragments [30]), was allowed to dry for 24h and then carefully removed from the leaves, and stored at -80°C.

#### *C. arabica—C. kahawae* samples

Hypocotyls of *C*. *arabica* (var. caturra) were inoculated with a conidial suspension of each *C*. *kahawae* isolate under study, as described above. To determine sampling times corresponding to the key time points of pathogenesis for the different *C*. *kahawae* isolates, the fungal pre-penetration, penetration and post-penetration stages were evaluated by light microscopy, as previously described [19, 30]. Plant material was harvested accordingly with the stages referred above: i) differentiation of melanized appressoria at 24 hours post inoculation (hpi) for all *C*. *kahawae* isolates; ii) fungal penetration and establishment of the biotrophic phase at 48 hpi for Ang29, Que2 and Zim12 but only at 72 hpi for Ang67; iii) switch to necrotrophy (onset of first symptoms) at 72 hpi for all isolates excluding Ang67, for which the first lesions only appeared at 96 hpi. Symptoms were recorded during the entire infection process, until the death of all hypocotyls, to confirm the aggressiveness profile of the isolates used.

Two independent experiments were conducted and 40 hypocotyls were collected for each isolate-time point combination. Hypocotyls were immediately frozen by immersion in liquid nitrogen and stored at -80°C.

### RNA extraction and cDNA synthesis

Total RNA was extracted with Spectrum™ Plant Total RNA Kit (Sigma-Aldrich, USA) according to the manufacturer’s instructions, for all samples. Residual genomic DNA was digested with DNase I (On-Column DNase I Digestion Set, Sigma-Aldrich, USA). Lyophilized samples of conidia and appressoria formed *in vitro* were grounded in the Lysis Solution with sand, while samples containing mycelium, nail polish with appressoria formed *in vivo* and infected hypocotyls were grounded in liquid nitrogen. RNA purity and concentration were measured at 260/280 nm and 260/230 nm using a spectrophotometer (NanoDrop-1000, Thermo Scientific, USA), while RNA integrity was verified by agarose gel electrophoresis. Genomic DNA contamination on the crude RNA samples was verified by qPCR analysis in an iQ5 real-time thermalcycler (Bio-Rad, USA), using EvaGreen® Supermix (Bio-Rad). Each 15 μl reaction comprised 5 μl of crude RNA, 7.5 μl EvaGreen Supermix and 200 nM of each *ck39066* primer. First-strand cDNA was synthesized from 1.0 μg of total RNA for *C*. *kahawae* samples and from 1.7 μg of total RNA for *C*. *arabica*-*C*. *kahawae* samples in a 20 μl final volume, using Omniscript RT kit (Qiagen, Germany) and Oligo(dT)_18_ primer (MBI Fermentas, Lithuania), following the manufacturer’s instructions. The cDNA was diluted 1:25 with sterile water for *C*. *kahawae* samples, and 1:20 for *C*. *arabica*-*C*. *kahawae* samples and stored at -20°C.

### Selection of candidate reference genes

The candidate RGs were selected based on two different approaches: three were selected from the literature as the most promising RGs for related plant pathogenic fungi; and five were selected from Illumina RNA-seq datasets, representing the early events of a compatible and an incompatible *Coffea* sp.- *C*. *kahawae* interaction (24, 48 and 72 hpi), based on gene expression stability [17]. The RGs retrieved from the literature were selected considering different functional classes, in order to reduce the chance of co-regulation and thus avoid a significant bias in geNorm analysis. These included: actin (*Act*) [31, 32]; cyclophilin type peptidyl-prolyl cis-trans isomerase precursor (*Cyp*) [15]; and serine threonine-protein phosphatase (*PP1*) [32]. For these genes it was necessary to obtain the DNA sequence for *C*. *kahawae* in order to design species specific qPCR primers. Novel sequences were lodged in GenBank with accession numbers KU579251 to KU579253.

For the Illumina RNA-seq-based approach, candidate RGs were selected among the 653 *C*. *kahawae* genes previously identified [17]. The candidate RGs were ranked according to the following criteria: presenting a fold change between sample collection times (24, 48, 72 hpi) close to one and low standard errors (tested pairs: 24/48 hpi, 48/72 hpi and 24/72 hpi for both compatible and incompatible interactions), i.e. with a constitutively expression along the infection period studied [17]. The top six ranked genes selected under these criteria were *ck20430* (predicted 60S ribosomal protein L18 gene); *ck28444* (predicted membrane biogenesis protein yop1); *ck36020* (predicted stf2-like protein); *ck48742* (predicted 40S ribosomal protein S28); and *ck34620* (homologue to hypothetical protein CGGC5_3535).

Specific primers (Table 2) were designed for each gene with PerlPrimer v1.1.17 [33]. Whenever possible, primers were designed in the junction of two different exons, thus preventing amplification of potential residual DNA. Amplicon size ranged between 80-200bp for all genes used, except for *PP1*, *cat2* and *thr1* for which longer fragments had to be produced (222- 269pb) in order to ensure the design of good quality primers (Table 2). The sequence of cDNA was obtained from the Illumina RNA-seq database [17] and the DNA sequence was inferred based on the alignment with sequences from other *Colletotrichum* species, and subsequently verified upon sequencing of the amplicon obtained for *C*. *kahawae* (S1 File).

**Table 2:** Detailed description of candidate reference genes, genes of interest, primer sets and qPCR amplification conditions.

Name	Description	Go terms Molecular Function	Primer sequence (5'-3')	Amplicon length (bp)	Annealing temperature (°C)	PCR efficiency	Primers designed in intron-exon boundary?
**Act**	**Actin**	**GO:0005524; GO:0005200**	**F—CAACATTGTCATGTCTGGTGG R–GTACTCCTGCTTGGAGATCC**	**202**	**63**	**1,902**	**YES, F**
**Cyp**	**Cyclophilin type peptidyl-prolyl cis-trans isomerase B precursor**	**GO:0003755**	**F—AAGACCGCTGAGAACTTCCG R–CTCGCCGTAGATGGACTTGC**	**153**	**64**	**1,91**	**YES, R**
**PP1**	**Serine threonine-protein phosphatase**	**GO:0004722**	**F- CACTGGTTGGAGCGAAAACGR–CAGGATCTGGAACGAGCAAAG**	**250**	**60**	**1,919**	**YES, R**
**ck20430**	**60s ribosomal protein L18**	**GO:0003735**	**F- AGAGACCAACAGCACCACAC R-CCACAAGCACAAGAAGCCC**	**118**	**60**	**1.923**	**YES, R**
**ck28444**	**membrane biogenesis protein yop1**	**GO:0034613; GO:0048309; GO:0071786; GO:1902408; GO:0051292; GO:0016192**	**F- TAACAACCTCGAGAAGCAGACC R- CGACCCAGTAAGTCAGCCAC**	**206**	**60**	**1.935**	**YES, R**
**ck48742**	**40S ribosomal protein S28**	**GO:0003735**	**F-ACCAGACCCGTTCCATCATCC R- CAAGATCCGTTCCTCGTAATGTCC**	**158**	**58**	**1.929**	**YES, F**
**ck36020**	**stf2-like protein**	**None**	**F-CCACGGCCCCAACGAGGAGGAT R- GAGGGCTGCAGCACGAAACATTAGG**	**140**	**60**	**1.930**	**NO**
**ck39066**	**homologue to hypothetical protein CGGC5_4189**	**None**	**F-AAGGGTGAATGGTTGAAGGG R- CTGCGTATGGGAAGAAGTAGAC**	**151**	**60**	**1.907**	**NO**
**ck34620**	**homologue to hypothetical protein CGGC5_3535**	**None**	**F-CCCGACTTCCACTTCCATTACC R-CGCCGACCAGGATGAACTTG**	**208**	**63**	**1.926**	**NO**
**ck21238**	**bifunctional catalase-peroxidase cat2**	**GO:0004096; GO:0004601; GO:0016491; GO:0020037; GO:0046872**	**F- TTCCGCATCTACCTTCCG R- TCAACACCAGCAACACCAC**	**222**	**60**	**1.950**	**NO**
**ck25805**	**trihydroxynaphthalene reductase**	**GO:0008152; GO:0055114**	**F- ATGTACCGTGATGTCTGCC R- GTGATCCAATCTACTAATACCAGCC**	**269**	**60**	**1.921**	**YES, F**

The specificity of real-time PCR products was confirmed by the presence of a single peak in the melting curve and the presence of a single band with the expected size upon 2% agarose gel electrophoresis stained, with GelRed nucleic acid staining (Biotium, USA).

### Quantitative real-time PCR

qPCR experiments were performed in an iQ5 real-time thermal cycler (Bio-Rad), using EvaGreen® Supermix (Bio-Rad). Each 15 μl reaction comprised 5 μl of the diluted cDNA, 7.5 μl EvaGreen Supermix, 200 nM of each primer and 1.7 μl of sterile distilled water. Thermal cycling for all genes was carried out under the following conditions: 3 min. of polymerase activation at 95°C; followed by 45 cycles of denaturation at 95°C for 10 s, and 30 s annealing at the annealing temperature for each gene (Table 2). A melting curve analysis was performed at the end of the PCR run over the range 55–95°C, increasing the temperature in a stepwise fashion by 0.5°C every 10s. Each set of reactions included a negative control with no template. Dissociation curves and agarose gel electrophoresis were used to analyze non-specific PCR products. The efficiency of each primer pair (amplification efficiency, E) was experimentally tested with the LinRegPCR program [34], which uses a linear regression analysis of fluorescence data from the exponential phase of PCR amplification to determine amplification efficiency (E). Two independent experiments comprising three biological replicates and two technical replicates were used for each sample.

### Assessment of gene expression stability

The expression stability of each candidate RG and the best combination of RGs were obtained using a pairwise method by geNorm [8] and a model-based method by NormFinder [9] software. The geNorm algorithm calculates the expression stability (M) based on the average pairwise variation between all RGs tested. The gene with the lowest M value is considered to have the most stable expression, while that with the highest M value presents a high variance in its expression [8]. Moreover, geNorm estimates the normalization factor (NF) using the geometric mean of expression levels of n best RGs, using a pairwise variation (V) with a cut-off value of 0.15. By contrast, NormFinder uses a model-based algorithm that takes into account the overall stability, as well as the stability of any groups that may be present in the sample set. This software ranks the stability values of candidate RGs, being the lowest stability value to the most stable expressed gene [9].

The analyses were performed considering two different datasets: i) all *C*. *arabica-C*. *kahawae* samples; and ii) all *C*. *kahawae* samples. Moreover, in order to test for differences between *C*. *kahawae* aggressiveness patterns, the *C*. *arabica—C*. *kahawae* samples were also analyzed separately according to the different isolates under study (Ang29, Ang67, Zim12 and Que2).

For *C*. *arabica—C*. *kahawae* samples a biomass correction step was required. This method, previously described for *Hemileia vastatrix* [35], takes into account the variation of fungal biomass within the samples across the infection process. Therefore, DNA was used to estimate the fungal biomass across key time points of pathogenesis. The DNA was extracted, from the same hypocotyls used in RNA assays, using the DNeasy Plant mini kit (Qiagen) as recommended by the manufacturer. Genomic DNA purity, concentration and integrity were determined as described above for RNA samples, and samples were diluted to 10ng.μl^-1^.

qPCR amplifications were performed using the same conditions previously described in section “Quantitative real-time PCR”, with the primer set *ck39066* (homologue to hypothetical protein CGGC5_4189)(Table 2). The Cq results were then used to normalize the Cq of RGs using the following formula:

Cqcorrected=CqcDNAreferencegenes−CqDNAck39066

It should be noted that this additional correction step is only required for the selection of stable RGs, not in the subsequent gene expression analysis. The Cq value of each RG obtained for all samples was transformed into relative quantity (Q) as compared to the Cq value of ungerminated conidia samples, using the formula previously described by Pfaffl *et al*. [36]. The definition of the optimal number of genes required for normalization was conducted by geNorm pairwise variation analysis [8]. A comprehensive ranking was established by calculating the arithmetic mean ranking value of each gene using the two applets, and each gene was ranked from 1 (most stable) to 8 (least stable) [37].

### Expression profiles of two genes of interest

The expression profile of two pathogenesis-related genes, previously described as being involved in host penetration of several fungi, was analyzed in this work to validate the RGs selected for each dataset. The target genes *cat2* and *thr1* encode a bifunctional catalase-peroxidase, involved in ROS detoxification [20–22, 26], and a trihydroxynaphthalene reductase, involved in the melanin biosynthesis pathway [23–25], respectively.

*Colletotrichum kahawae* genes homologous to *cat2* and *thr1* were retrieved from the *C*. *kahawae* RNA-seq database, showing differential expression in the early phases of the infection process (unpublished data). Specific primers (Table 2) were designed with PerlPrimer v1.1.17 [33], as described above.

To assess gene expression, relative quantities (RQ) were calculated for both (RGs and genes of interest) using the formula *RQ* = *E*^Δ*Cq*^ where E represents the amplification efficiency (E) for each gene and ΔCq the difference between the Cq from each target sample and the conidia sample (Δ*Cq* = *Cq conidia* − *Cq target*) [36]. A normalization factor (NF), calculated as the geometric mean of the relative quantities of the RGs selected for each normalization approach was used to obtain the normalized relative quantities [36]. In this work, six different normalization factors were tested: i) best three ranking genes selected for *C*. *arabica*—*C*. *kahawae* samples (**NF Global** *C*. *arabica—C*. *kahawae* interaction); ii) best normalization factor identified by geNorm for both datasets (**NF Best geNorm**); iii) best three ranking genes selected for *C*. *arabica—C*. *kahawae* samples according to the different *C*. *kahawae* isolates under study (**NF Best aggressiveness pattern**); iv) best two ranking genes common to both datasets (**NF Best two ranking genes)**; v) best three ranking genes selected for C. *kahawae* samples (**NF Global** *C*. *kahawae*); vi) worst ranking genes selected for both datasets (**NF Worst**).

Statistically significant differences between the six normalization factors used were determined by the Kruskall-Wallis test. Potential differences between the time points *for C*.*arabica-C*.*kahawae* and between lifecycle stages for *C*. *kahawae* were tested using the Mann-Whitney test. Both tests were computed in IBM® SPSS® Statistics version 20.0.0 (SPSS Inc., USA) software and they were considered significant when p < 0.05.

## Results and Discussion

Reference gene validation for qPCR expression studies has become a fundamental requisite for reliable quantification results. Here, we describe an assessment of eight candidate RGs for their use as internal controls in gene expression studies of *C*. *kahawae* and *C*. *arabica—C*. *kahawae* interaction samples. In this work, two different approaches were applied to identify suitable candidate RGs: analysis of traditional RGs previously validated for other fungi (literature); and of new candidate RGs from Illumina RNA-seq datasets. The genes selected from the literature included *Act*, *Cyp*, and *PP1*, while the genes selected from Illumina RNA-seq datasets were *ck20430*, *ck28444*, *ck36020*, *ck48742* and *ck34620* (Table 2).

### Amplification specificity and efficiency

To investigate the expression stability of the candidate RGs selected, transcript levels of the eight candidates were measured by qPCR using gene-specific primer pairs (Table 2). Specificity of real-time PCR products was confirmed by the presence of a single peak in the melting curve and the presence of a single band with the expected size after agarose gel electrophoresis (S2 File.). No amplification was obtained for the negative control. The average PCR efficiency of primers ranged from 1.902 to 1.950 (Table 2) as calculated by LinRegPCR [34]. Considering that the efficiency value for one primer pair could be different among the type of samples (*C*. *kahawae* samples or *C*. *arabica-C*. *kahawae* samples), a separate analysis was performed, but no significant differences were observed (S1 Table).

### Determination of Cq values and variation on candidate RGs

**Fig 1:**
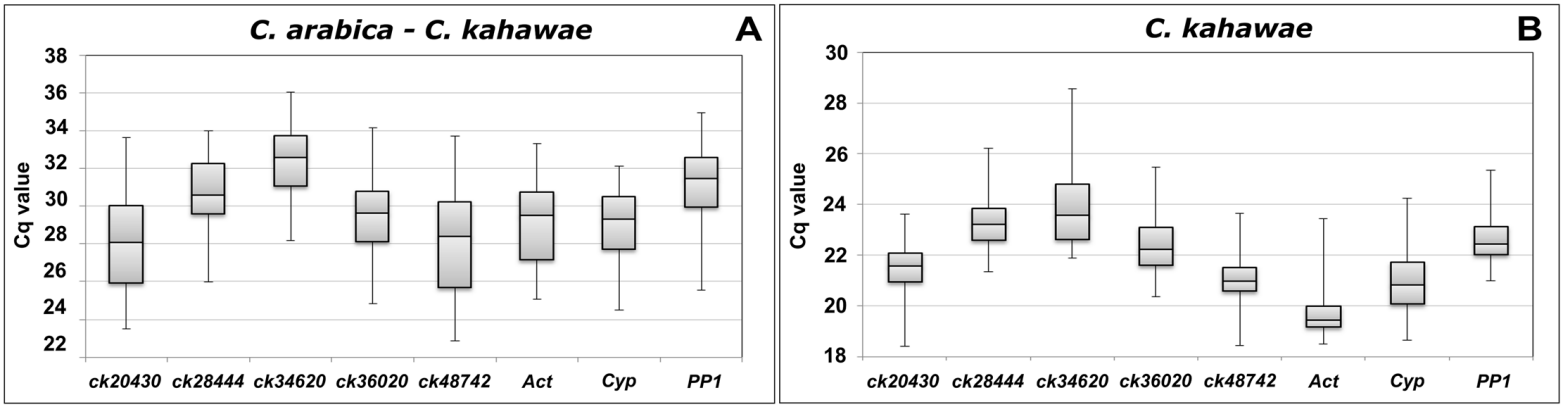
Box and whisker plots of Cq values for each reference gene across the experimental samples. A) *C*. *arabica-C*. *kahawae* samples; B) *C*. *kahawae* samples. The boxes indicate the 25th and 75th percentiles. Lines within the boxes represent the median Cq values; the whiskers mark minimum and maximum values in each data set.

Following PCR amplification, a general overview of the expression profile and relative abundance of each candidate gene was obtained by plotting the Cq values obtained for *C*. *arabica-C*. *kahawae* samples as well as for *C*. *kahawae* samples (Fig 1).

Most candidate RGs displayed median Cq values for fungus samples ranging from 19 to 23, indicating a moderately high level of expression. However, in *C*. *arabica-C*. *kahawae* samples, the median Cq for the same candidate RGs ranged from 28 to 32. The minimum Cq values, meaning higher abundance, were observed for *Act* (18.5) in mature appressoria formed after 18–22 h on polystyrene, while *ck34620* had the lowest expression value (35.5) on the early stages of the infection process. Overall, gene expression variation across samples taken from different stages of the infection process ranged from 7.6 to 10.9 Cq, while for C. *kahawae* samples ranged only between 4.4 and 6.7 Cqs (Fig 1). These changes in Cq values may reflect the variations in the amount of fungal RNA present in *C*. *arabica—C*. *kahawae* interaction samples at each time point. According to previous studies [35], the effect of fungal biomass variation in *C*. *arabica*—*C*. *kahawae* samples can be corrected using the Cq value of the amplification of a gene from DNA extracted from the same samples used for RNA quantification. In spite of this, occasionally, the Cq value for each candidate RG did not followed a strictly parallel line to that of genomic DNA, suggesting variations in the expression levels of candidate RGs (S1 Fig). Though preliminary information can be obtained through absolute Cq analysis (Fig 1), to correctly assess the expression stability of candidate genes, it is crucial to use statistical tools like geNorm and NormFinder to determine the best set of RGs.

However, since these statistical tools were developed to assess the stability of RGs when the amount of RNA is similar under studied conditions [8, 9], a correction step was applied to allow an accurate analysis of candidate RGs stability in *C*. *arabica*—*C*. *kahawae* samples.

### Analysis of gene expression stability data

The expression stability of the candidate RGs, as well as the number of RGs necessary for accurate gene-expression profiling, was performed using two different statistical applets, geNorm [8] and NormFinder [9]. Though both aim to determine which candidate RGs are the most stable under certain conditions, they run under different algorithms and mathematical models. Therefore, the stability ranking of the putative RGs might differ depending on the software used as previously described [18, 38]. The results were analyzed dividing the data into two different datasets: i) all *C*. *arabica—C*. *kahawae* samples; ii) all *C*. *kahawae* samples. Moreover, the datasets of *C*. *arabica—C*. *kahawae* samples were analyzed separately considering the different fungal isolates under study (Ang29, Ang67, Zim12 and Que2) to check if the set of candidate RGs changes according to the interaction established.

As shown in Table 3 slight differences were found between the stability rankings of RGs provided by the two computational programs for the *C*. *arabica—C*. *kahawae* samples, being the *PP1*, ck34620 and *Act* selected as the most stable genes. By contrast, in the analysis of *C*. *kahawae* samples, the best three selected RGs changed according to the software used. In the geNorm analysis, the best set of candidate RGs were *ck20430*, *ck48742* and *PP1*, while in the NormFinder analysis the selected set of RGs were *PP1*, *Act* and *ck20430*, followed by *ck48742*. This incongruence was previously described in other RG validation studies [18, 38, 39] and reveals the importance of using a comprehensive ranking analysis. Currently several strategies exist to create a comprehensive stability ranking which integrate the results of the two software. Here, a certain weight was assigned to each gene corresponding to the rank obtained from each program (e.g. 1- most stable to 8-least stable). Subsequently, the rank aggregation relied on straightforward arithmetic and geometric means of the ranks (Table 3) [37]. Taking into account the comprehensive ranking results, the top three most stable genes for *C*. *arabica—C*. *kahawae* samples were *PP1*, *ck34620* and *Act*, while for *C*. *kahawae* samples were *PP1*, *Act* and *ck20430* (Table 4). Although the best set of RGs changed according to the type of samples, *PP1* and *Act* seemed to be stable under all tested conditions. The higher stability of *Act* and *PP1* was previously described for *Beauveria bassiana* under all analyzed conditions [32]. However, for other fungi such as *Metarhizium anisopliae*, *Act* was not among the most stable RGs [31], which reinforce the importance of developing these studies.

**Table 3:** Comprehensive ranking of candidate reference genes for each of the datasets used: i) all *C*. *arabica*-*C*. *kahawae* interaction samples; ii) all *C*. *kahawae* samples.

	Global *C. arabica-C. kahawae*					Global *C. kahawae*				
Gene Name	NormFinder		geNorm		Overall ranking	NormFinder		geNorm		Overall ranking
	Stability value	Ranking	M value	Ranking		Stability value	Ranking	M value	Ranking	
***ck48742***	0.664318499	8	1.678009	7	**8**	0.369507389	4	0.512228	1	**3**
***ck20430***	0.620810946	7	1.614348	6	**7**	0.340416201	3	0.512228	1	**2**
***ck36020***	0.567729528	6	1.482661	5	**6**	0.59947532	8	1.361478	7	**8**
***ck28444***	0.547208462	5	1.369675	3	**4**	0.381965941	5	0.986202	4	**5**
***Cyp***	0.527026519	4	1.421997	4	**4**	0.386556712	6	1.047293	5	**6**
***Act***	0.50941143	3	1.153881	1	**3**	0.258646654	2	0.891739	3	**3**
***ck34620***	0.433946008	2	1.153881	1	**1**	0.465745858	7	1.167152	6	**7**
***PP1***	0.265506703	1	1.283251	2	**1**	0.24107307	1	0.775031	2	**1**

**Table 4:** Normalization factors tested for gene expression analysis referring to the candidate reference genes included for each sample type.

Normalization factors	*C. arabica-C. kahawae*				*C. kahawae*
^**1**^**NF Global** *C*. *arabica-C*. *kahawae*	*PP1; Act; ck34620*				*PP1; Act; ck34620*
^**2**^**NF Best geNorm**	*PP1; Act; ck34620; ck28444; Cyp*				*ck48742; Act; PP1; ck20430*
^**3**^**NF Two Best ranking genes**	*PP1; Act*				*PP1; Act*
^**4**^**NF Global** *C*. *kahawae*	*PP1; Act; ck20430*				*PP1; Act; ck20430*
^**5**^**NF Worst**	*ck20430; ck48742; ck36020*				*ck34620; ck36020*
^**6**^**NF Best aggressiveness pattern**	**Ang29**	**Ang67**	**Zim12**	**Que2**	** **
	*Cyp; Act; ck28444*	*Cyp; Act; ck34620*	*Cyp; Act; PP1*	*Cyp; Act; PP1*	** **

^1^ The best three ranking genes selected for *C*. *arabica*—*C*. *kahawae* samples;

^2^ The best normalization factor identified by geNorm for both datasets;

^3^ The best two ranking genes common to both datasets

^4^ The best three ranking genes selected for C. *kahawae* samples

^5^ The worst ranking genes selected for both datasets

^6^The best three ranking genes selected for *C*. *arabica—C*. *kahawae* samples according to the different isolates under study.

**Fig 2:**
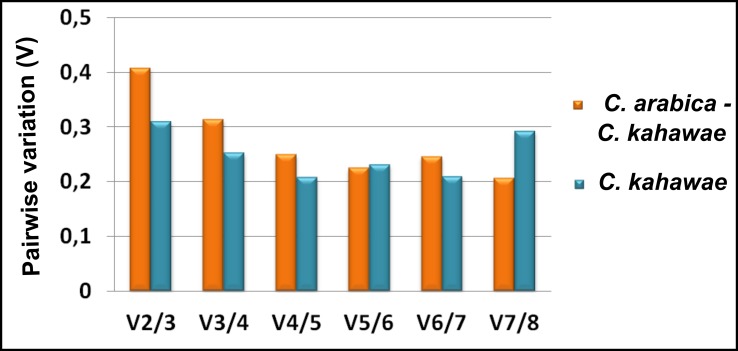
Prediction of the optimal number of reference genes required for effective normalization. Pairwise variation (V) of the candidate reference genes calculated by geNorm using the two different datasets studied: i) all *C*. *arabica-C*. *kahawae* samples; ii) all *C*. *kahawae* samples.

The analysis carried out by geNorm enabled the determination of the optimal number of RGs through the calculation of pairwise variation (Vn/Vn+1) between two sequential candidate RGs. High values indicate the need for the inclusion of another gene to obtain a reliable normalization factor, which should contain at least two RGs. Thus, extra RGs can be included until the Vn/Vn+1 is smaller than a threshold of 0.15, as recommended [8]. However, this is not an absolute value and it can change according to the data [8]. As shown in Fig 2, the pairwise variation values, for both datasets, are higher than the recommended cut-off value. Based on this parameter, the use of five reference genes (V5/6 = 0.224248) for the *C*. *arabica*—*C*. *kahawae* sample dataset and four (V4/5 = 0.207316) for the fungal dataset seems to be the best approach (lowest value). Therefore, the **NF Best geNorm** for *C*. *arabica*—*C*. *kahawae* samples comprises the use of *PP1; Act; ck34620; ck28444; Cyp* while the **NF Best geNorm** for *C*. *kahawae* samples includes *ck48742; Act; PP1; ck20430* as best reference genes. However it is crucial to validate if the use of five/four genes is indeed necessary to obtain a proper data normalization, or if this purpose can be reached with only two or three RGs, since a trade-off between the practical considerations, such as time/cost, and accuracy, should be considered, especially if no significant differences are observed.

A separate analysis of the *C*. *arabica*—*C*. *kahawae* samples according to the different isolates under study was further performed, in order to test for significant differences related with aggressiveness patterns. Despite the slight differences between the results provided by geNorm and NormFinder, the same best three RGs (*Act*, *Cyp* and *PP1*) were identified for Zim12 and Que2, while for Ang29 the best set was *Act*, *Cyp* and *ck28444*, and for Ang67 *Act*, *Cyp* and *ck34620*, being *PP1* the fourth most stable RG for the latter isolate (S2 Table). Although these results point to a different conclusion from the global approach, almost all genes showed a stability value lower than the recommended cut-off value of 1.5 [8], noting their high stability. However, a proper validation of the selected candidate RGs will be crucial to prove if these differences on the Normalization factor used can significantly change the expression of target genes.

The worst candidate RGs for *C*. *kahawae* samples were *ck34620* and *ck36020*, while for *C*. *arabica*—*C*. *kahawae* samples were *ck36020*, *ck48742* and *ck20430*. Although all least stable genes were candidates retrieved from the RNA-seq datasets, these are nevertheless a promising source of additional (and potentially independent) qPCR reference genes, since, for in both types of samples (*C*. *kahawae* and *C*. *arabica-C*. *kahawae* interactions), the best set of reference genes (Tables 3 and 4) included genes from the literature and genes from RNA-seq data. Similar results were observed in a previous study involving *Fusarium graminearum* [15]. Moreover, the current RNA-seq dataset is derived from *C*. *kahawae*-infected coffee leaves, therefore comprising a mixture of plant and fungal RNA which restricts the number of pathogen genes retrieved. Still, this dataset was sufficient to generate five candidate RGs, suggesting that larger datasets might provide higher numbers of candidate RGs.

### Expression analysis of pathogenesis-related genes

Two different genes of interest (*cat2* and *thr1*), related with the early stages of the infection process, were used to validate the best normalization factor for qPCR data analysis of *C*. *kahawae*, regarding its aggressiveness pattern or developmental stages, using the conidia as control.

Six different normalization factors were tested, according to the results obtained by geNorm and NormFinder, in order to correctly choose the best set of reference genes as shown in Table 4: i) **NF Global** *C*. *arabica—C*. *kahawae* interaction; ii) **NF Best geNorm**; iii) **NF Best aggressiveness pattern**; iv) **NF Best two ranking genes**; v) **NF Global** *C*. *kahawae*; vi) **NF Worst**.

Considering the normalization factors tested, the expression profile of *thr1* showed only slight differences between most of them (S2 Fig and S3 Fig), being the worst normalization factor the exception. For the *C*. *arabica*—*C*. *kahawae* dataset, no significant differences were observed between the first five NFs, when the Kruskal-Wallis test was applied (S3 Table). However, significant differences were observed between these and the worst normalization factor (S3 Table). For the fungal datasets a similar result were observed (S4 Table).

The expression profile of *cat2* only changed drastically when the worst normalization factor was applied (S2 Fig and S3 Fig), but significant statistical differences were observed between the different normalization factors tested (S5 Table). When applying NF Best geNorm, which includes the five best genes selected for the *C*. *arabica—C*. *kahawae* dataset, as well as the four best genes selected for the *C*. *kahawae* dataset, no significant differences in expression levels were detected in comparison with the use of NF Global *C*. *arabica—C*. *kahawae* or NF Global *C*. *kahawae* (S5 and S6 Tables), with only 3 reference genes. Thus, adding two or one more reference genes did not increased the accuracy of the results. Moreover, no significant differences were observed when comparing NF Best aggressiveness pattern with NF Global *C*. *arabica*—*C*. *kahawae* or NF Best geNorm (S5 Table). These results show that the same normalization factor could be used to normalize all the isolates regardless of its aggressiveness pattern. In contrast, the best two ranking genes seem to have significant differences, when compared to NF Best geNorm, NF Best aggressiveness pattern or NF Global *C*. *arabica*—*C*. *kahawae* (S5 Table). These results shows that, although *PP1* and *Act* genes appear to be stable under all conditions studied, the use of only these two RGs is not the best approach for an accurate normalization approach. Finally, the worst normalization factor has significant differences when compared with the five normalization factors previously described for both datasets (S5 and S6 Tables).

**Fig 3:**
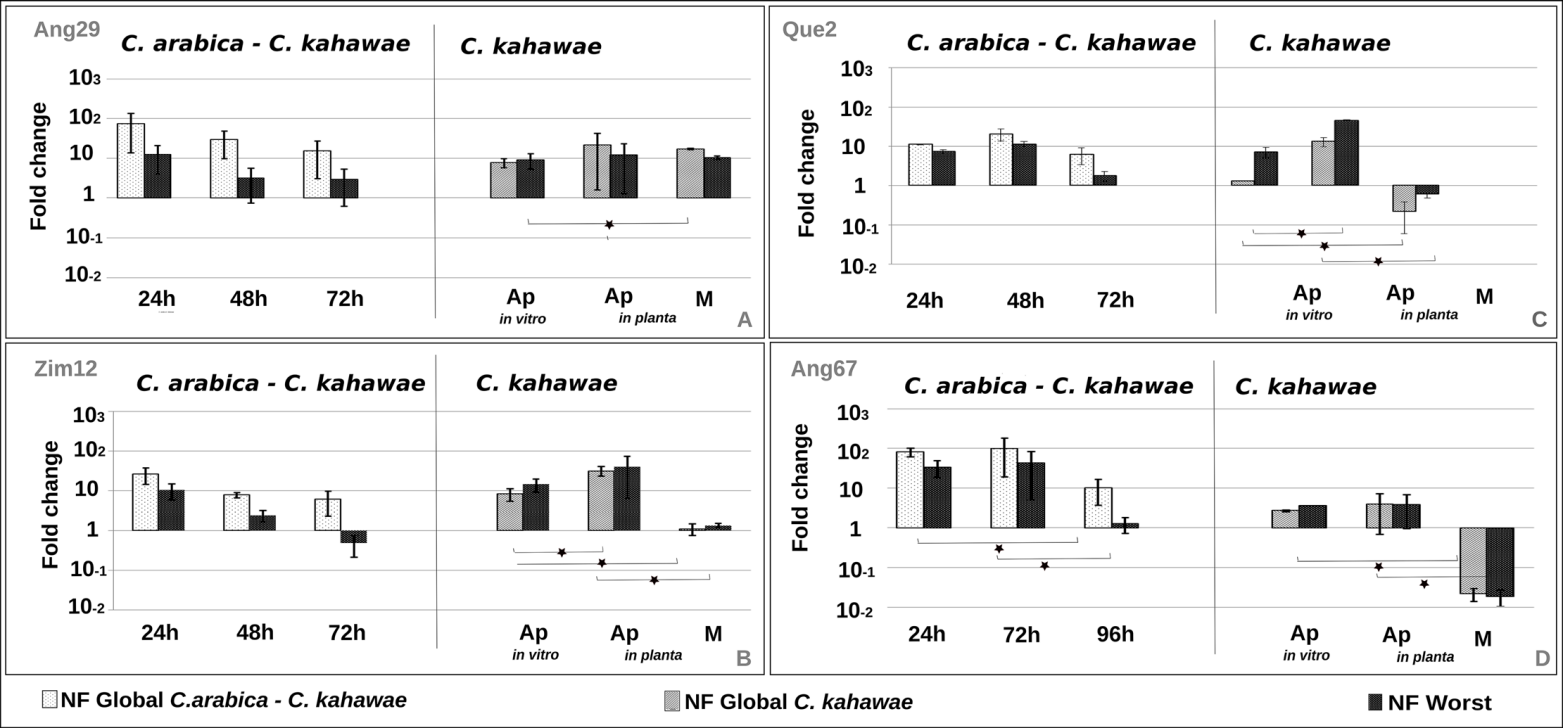
Relative quantification of *thr1* expression using the Best and the Worst normalization factors (NF). Expression profiles are presented per isolate (Ang29 (A), Zim 12 (B), Que2 (C) and Ang67 (D)), during the early stages of infection process and growth (Ap: Appressoria; M: Mycelium). The *C*. *arabica–C*. *kahawae* samples were normalized with **NF Global** *C*. *arabica—C*. *kahawae* interaction (*PP1; Act; ck34620)* and **NF Worst** (*ck20430; ck48742; ck36020)*, while the *C*. *kahawae* samples were normalized with **NF Global** *C*. *Kahawae* (*PP1; Act; ck20430*) and **NF Worst** (*ck34620; ck36020*).

**Fig 4:**
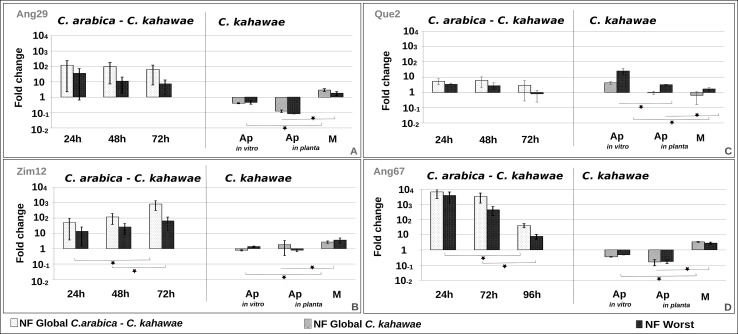
Relative quantification of *cat2* expression using the best and the Worst normalization factors (NF). Expression profiles are presented per isolate (Ang29 (A), Zim 12 (B), Que2 (C) and Ang67 (D)), during the early stages of infection process and growth (Ap: Appressoria; M: Mycelium). The *C*. *arabica–C*. *kahawae* samples were normalized with **NF Global** *C*. *arabica—C*. *kahawae* interaction (*PP1; Act; ck34620)* and **NF Worst** (*ck20430; ck48742; ck36020)*, while the *C*. *kahawae* samples were normalized with **NF Global** *C*. *Kahawae* (*PP1; Act; ck20430*) and **NF Worst** (*ck34620; ck36020*).

In summary, under the present experimental system, the best normalization factor for *C*. *arabica*—*C*. *kahawae* interaction samples comprises the use of *PP1*, *Act* and *ck34620*, while for *C*. *kahawae* samples the best normalization factor is provided by the joint use of *PP1*, *Act* and *ck20430*. Moreover, the significant differences observed on the expression profile of the genes of interest when normalized with the Best or the Worst NF shows the need to validate the best set of reference genes for each specific experimental condition (Fig 3 and Fig 4). Such, sample-specific normalization requisites were previously described for many other organisms, namely *Hemileia vastatrix*, *Fusarium graminearum*, *Vitis vinifera* and *Caragana intermedia* [12, 15, 35, 38].

The expression profiles of *cat2* and *thr1* during the infection time-course (*C*. *arabica*—*C*. *kahawae* samples) were similar, showing a highest expression peak in the early stages of the infection process (24/48h) followed by a slight decrease over time (Figs 3 and 4). However, significant differences on the expression between the time points were only statistical significant for the Ang 67 isolate (S7 and S8 Tables). In contrast, the expression profile of *Cat2* for Zim 12 isolate had a significant slight increase on the expression over the time (Fig 4, S7 and S8 Tables). Previous studies on *Colletotrichum acutatum* showed a high level of expression for *cat2* during appressoria formation when compared with mycelia grown under nitrogen limitation or a complete nutrient supply [21]. Therefore, the high expression levels detected in the early stages of infection process were expected. Further studies will be required to understand the isolate-specific expression pattern of *Cat2* observed for Zim 12.

On the other hand, for the *C*. *kahawae* samples, different expression profiles were observed between the two genes. *cat2* seemed to be expressed only in the mycelium and repressed in appressoria, both *in planta* and *in vitro*, with significant differences (Fig 4), while *thr1* expression was higher *in planta* and *in vitro* appressoria and was repressed on mycellium for almost all isolates, with significant differences (Fig 3). For Ang 29 the *thr1* gene seems to be highly expressed in all *C*. *kahawae* samples with only significant difference between Ap *in vitro* and mycellium (S7 and S8 Tables). Previous studies on *Colletotrichum lagenarium* showed an increase on the expression of *thr1* during spore germination [23].

Despite the interesting expression profile of these genes, subsequent targeted expression studies will be required to associate different expression profiles with the aggressiveness patterns of the isolates.

## Conclusions

In the present study, we evaluated the expression stability of eight candidate reference genes across several *C*. *kahawae* samples representing different growth and infection stages, with the aim of identifying the best set for data normalization of gene expression studies. New candidate reference genes were selected based on a genome wide approach (RNA-seq datasets) and among them most had expression stability similar to the typical reference genes selected in the literature. This highlights RNA-seq data as alternative sources of reference genes, in addition to classical (i.e., literature-based) reference genes. Two main normalization factors were selected according to the type of samples under study, applying a combination of reference genes: *PP1*, *Act* and *ck34620* were the best set of reference genes when *C*. *arabica*—*C*. *kahawae* samples were used, while *PP1*, *Act* and *ck20430* were the best set when only *C*. *kahawae* samples were used. Unfortunately, although *PP1* and *Act* were the two most stably expressed RGs, the NF common to both type of samples, relying only on these RGs, does not seem to constitute a good normalization factor for all tested samples. The expression profiles of *cat2* and *thr1* during the infection time-course on *C*. *arabica*—*C*. *kahawae* samples were similar, with the highest expression peak in the early stages of the infection process 24/48 hpi and a slight decrease over time. For *C*. *kahawae* samples, different expression profiles were observed between the two genes, being cat2 more expressed in the mycelium and repressed in appressoria, while *thr1* expression was higher *in planta* and *in vitro* appressoria. This work provides the first reference genes specifically established for *C*. *kahawae* samples, and this information will greatly facilitate future studies of gene expression in *C*. *kahawae*.

## Supporting Information

S1 Fig Cq values of reference genes compared with fungal biomass normalization. RNA transcription levels of candidate reference genes tested during the infection time-course are presented as Cq mean value in the different samples, against the respective biomass quantification with Cq DNA value (*ck39066*), for two independent experiments.
(TIF)

S2 Fig Relative quantification of *thr1* expression using six different normalization factors (NF). Expression profiles are presented per isolate (Ang29 (A), Zim 12 (B), Que2 (C) and Ang67 (D)), during the early stages of infection process and growth (Ap: Appressoria; M: Mycelium). Details on the normalization factors are described in Table 4.
(TIF)

S3 Fig Relative quantification of *cat2* expression using six different normalization factors (NF). Expression profiles are presented per isolate (Ang29 (A), Zim 12 (B), Que2 (C) and Ang67 (D)), during the early stages of infection process and growth (Ap: Appressoria; M: Mycelium). Details on the normalization factors are described in Table 4.
(TIF)

S1 File cDNA sequences for the genes under study. (DOCX)

S2 File Primer specificity test through dissociation curve analysis collected from iQ5 (Bio-rad) using several samples of *C*. *kahawae* and *C*. *arabica–C*. *kahawae*. (DOC)

S1 Table Primer efficiency specific for the type of samples under study (*C*. *arabica-C*. *kahawae* samples and *C*. *kahawae* samples). (XLSX)

S2 Table Ranking of the candidate reference genes for the *C*. *arabica-C*. *kahawae* samples according to the isolates under study. Stability values and ranking of candidate reference genes given by geNorm and Normfinder are provided alongside with the overall ranking calculated by the arithmetic mean ranking value of each gene using the two applets. Genes were ranked from the most stable (1) to the least stable (8).
(XLSX)

S3 Table Statistical analysis of *thr1* expression in *C*. *arabica-C*. *kahawae* samples relative to the application of different normalization factors. Main statistics given by the statistic test Kruskal-Wallis on *thr1* expression for *C*. *arabica-C*. *kahawae* samples, comparing the normalization factors followed.
(XLSX)

S4 Table Statistical analysis of thr1 expression in *C*. *kahawae* samples relative to the application of different normalization factors. Test statistics given by the Kruskal-Wallis statistic onthre expression for *C*. *kahawae* samples, comparing the normalization factorsfollowed.
(XLSX)

S5 Table Statistical analysis of *cat2* expression in *C*. *arabica-C*. *kahawae* samples relative to the application of different normalization factors. Test statistics given by the Kruskal-Wallis statistic test on *cat2* expression for *C*. *arabica-C*. *kahawae* samples, comparing the normalization factors followed.
(XLSX)

S6 Table Statistical analysis of *cat2* expression in *C*. *kahawae* samples relative to the application of different normalization factors. Test statistics given by the Kruskal-Wallis statistic test on *cat2* expression for *C*. *kahawae* samples, comparing the normalization factors followed.
(XLSX)

S7 Table Statistical analysis of *thr1* expression between different time points. Test statistics given by the Mann-Whitney test on *thr1* expression, comparing time points *for C*.*arabica-C*.*kahawae* and lifecycle stages for *C*. *kahawae*.
(XLSX)

S8 Table Statistical analysis of *cat2* expression between different time points. Test statistics given by the Mann-Whitney test on *cat2* expression, comparing time points *for C*.*arabica-C*.*kahawae* and lifecycle stages for *C*. *kahawae*.
(XLSX)
