# Supplementary figures and images for: Comparative Validation of Conventional and RNA-Seq Data-Derived Reference Genes for qPCR Expression Studies of Colletotrichum kahawae
Source: PLoS One. 2016 Mar 7;11(3):e0150651. doi: 10.1371/journal.pone.0150651 (PMC4780792; doi:10.1371/journal.pone.0150651)

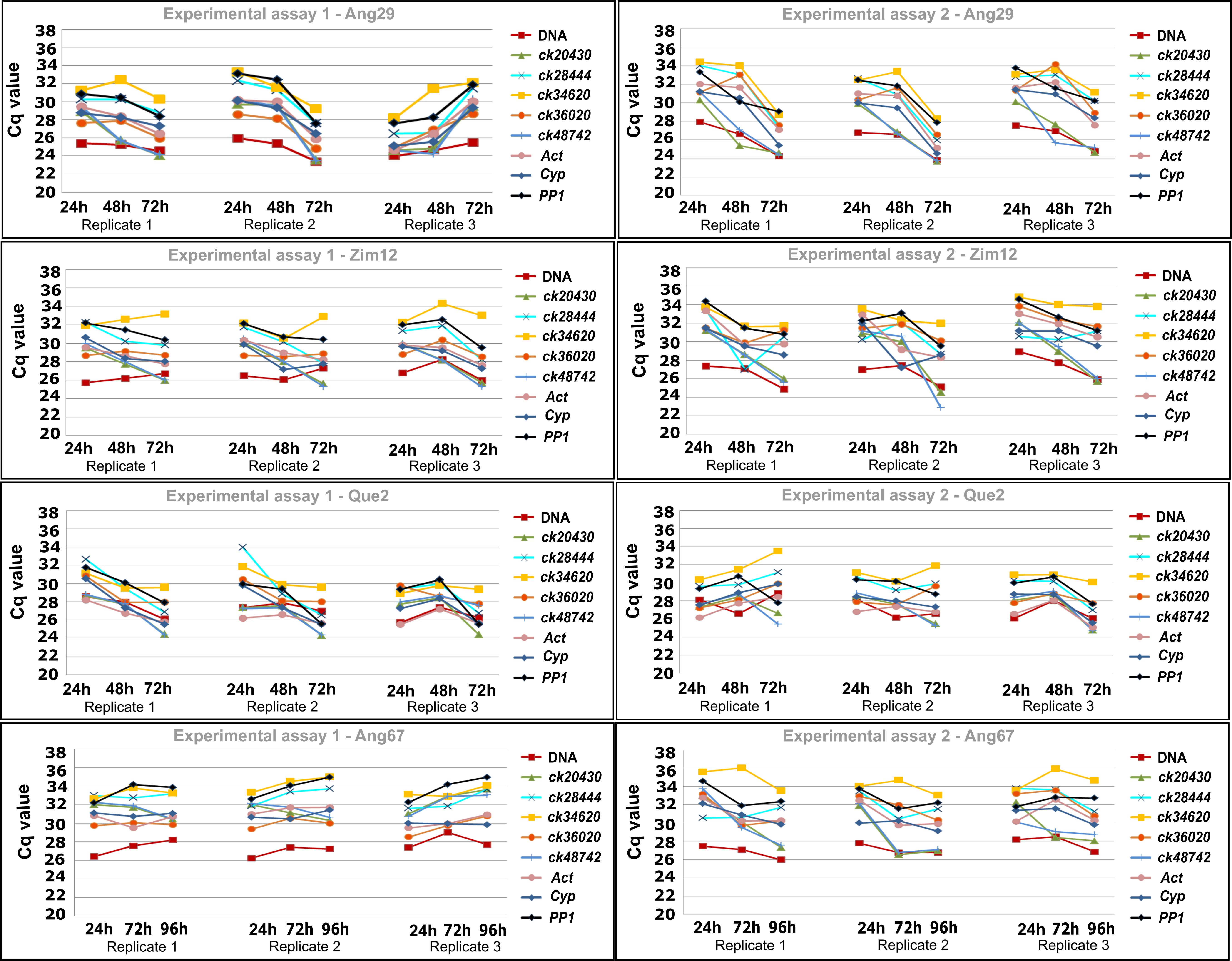

Supplement: S1 Fig — RNA transcription levels of candidate reference genes tested during the infection time-course are presented as Cq mean value in the different samples, against the respective biomass quantification with Cq DNA value (ck39066), for two independent experiments. (TIF) [file pone.0150651.s001.tif]
