# Supplementary material for: Comparative Validation of Conventional and RNA-Seq Data-Derived Reference Genes for qPCR Expression Studies of Colletotrichum kahawae
Source: PLoS One. 2016 Mar 7;11(3):e0150651. doi: 10.1371/journal.pone.0150651 (PMC4780792; doi:10.1371/journal.pone.0150651)
Supplement: S1 File — (DOCX) [file pone.0150651.s004.docx]

**S1 File. – cDNA sequences for the genes under study.**

**LOCUS Act** 202 bp mRNA linear

DEFINITION Colletotrichum kahawae strain CIFC Que2 actin (act) gene, mRNA partial sequence

SOURCE Colletotrichum kahawae (anamorph: Colletotrichum kahawae)

FEATURES Location/Qualifiers

source 1..202

/organism="Colletotrichum kahawae"

/mol_type="mRNA"

/host="Coffea arabica"

/country="Kenya"

/anamorph="Colletotrichum kahawae"

CDS <1..>202

/codon_start=2

/product="Actin"

/translation="NIVMSGGTTMYPGLSDRMQKEITSLAPSSMKVKIIAPPERKYSV

WIGGSILASLSTFQQMWISKQEY"

BASE COUNT 39 a 63 c 44 g 56 t

ORIGIN

1 caacattgtc atgtctggtg gtaccaccat gtaccctggt ctctccgacc gtatgcagaa

61 ggagatcact tctcttgctc cttcttccat gaaggtcaag atcatcgctc cccccgagcg

121 caagtactcc gtctggatcg gtggttccat tctggcttcc ctttctacct tccagcagat

181 gtggatctcc aagcaggagt ac

//

**LOCUS Cyp** 153 bp mRNA linear

DEFINITION Colletotrichum kahawae strain CIFC Que2 cyclophilin type peptidyl-prolyl cis-trans isomerase precursor (cyp) gene, mRNA partial sequence.

SOURCE Colletotrichum kahawae (anamorph: Colletotrichum kahawae)

FEATURES Location/Qualifiers

source 1..153

/organism="Colletotrichum kahawae"

/mol_type="mRNA"

/host="Coffea arabica"

/country="Kenya"

/anamorph="Colletotrichum kahawae"

CDS <1..>153

/codon_start=1

/product="cyclophilin type peptidyl-prolyl cis-trans isomerase precursor (cyp)"

/translation="KTAENFRALATGEKGFGYEGSTFHRVIKQFMIQGGDFTKGDGTG

GKSIYGE"

BASE COUNT 33 a 47 c 46 g 27 t

ORIGIN

1 aagaccgctg agaacttccg cgctctcgcc accggcgaga agggcttcgg ctacgaggga

61 tctaccttcc accgtgttat caagcagttc atgatccagg gcggtgactt caccaagggc

121 gacggcaccg gtggcaagtc catctacggc gag

//

**LOCUS PP1** 250 bp mRNA linear

DEFINITION Colletotrichum kahawae strain CIFC Que2 serine threonine-protein phosphatase (pp1) gene, mRNA partial sequence.

SOURCE Colletotrichum kahawae (anamorph: Colletotrichum kahawae)

ORGANISM Colletotrichum kahawae

FEATURES Location/Qualifiers

source 1..250

/organism="Colletotrichum kahawae"

/mol_type="mRNA"

/host="Coffea arabica"

/country="Kenya"

/anamorph="Colletotrichum kahawae"

CDS <1..>250

/codon_start=2

/product=" serine threonine-protein phosphatase (pp1)"

/translation="TGWSENDRGVSFTFGPDVVSRFLQKHDMDLICRAHQVVEDGYEF

FSKRQLVTLFSAPNYCGEFDNAGAMMSVDESLLCSFQIL"

BASE COUNT 50 a 70 c 67 g 63 t

ORIGIN

1 cactggttgg agcgaaaacg acagaggtgt ttccttcacc tttggtccgg acgttgtttc

61 ccgcttcttg cagaagcacg acatggactt gatctgccgt gcccatcagg tcgtcgagga

121 cggctacgag ttcttctcca agcgccaact tgttaccttg ttcagcgcac ccaactactg

181 cggagagttc gacaatgctg gagccatgat gagcgtagac gagagcctgc tttgctcgtt

241 ccagatcctg

//

**LOCUS ck20430** 118 bp mRNA linear

DEFINITION Colletotrichum kahawae strain CIFC Que2 60s ribosomal protein L18, mRNA partial sequence.

SOURCE Colletotrichum kahawae (anamorph: Colletotrichum kahawae)

FEATURES Location/Qualifiers

source 1..118

/organism="Colletotrichum kahawae"

/mol_type="mRNA"

/host="Coffea arabica"

/country="Kenya"

/anamorph="Colletotrichum kahawae"

CDS <1..91

/codon_start=2

/product="60s ribosomal protein l18"

/translation="HKHKKPYVESKGRKFERARGRRRSRGFKV"

BASE COUNT 22 a 34 c 37 g 25 t

ORIGIN

1 ccacaagcac aagaagccct acgtcgagtc caagggccgc aagttcgagc gtgcccgtgg

61 tcgcagacgc tctcgcggct tcaaggtcta aacgggttgt gtggtgctgt tggtctct

//

**LOCUS ck28444** 206 bp mRNA linear

DEFINITION Colletotrichum kahawae strain CIFC Que2 membrane biogenesis protein yop1, mRNA partial sequence.

SOURCE Colletotrichum kahawae (anamorph: Colletotrichum kahawae)

FEATURES Location/Qualifiers

source 1..206

/organism="Colletotrichum kahawae"

/mol_type="mRNA"

/host="Coffea arabica"

/country="Kenya"

/anamorph="Colletotrichum kahawae"

CDS <1..>206

/codon_start=2

/product="membrane biogenesis protein yop1"

/translation="NNLEKQTNVPKAYAVIGVAALYFFLIIFNLGGQLLTNIAGFVIP

GYYSLNALFTANKQDDTQWLTYWV"

BASE COUNT 42 a 70 c 45 g 49 t

ORIGIN

1 taacaacctc gagaagcaga ccaatgttcc caaggcctac gccgtcatcg gtgtcgccgc

61 cctctacttc tttctcatca tcttcaacct cggcggtcag cttctgacca acattgcggg

121 cttcgtgatt cccggttact actccctgaa cgccctgttc actgcgaaca agcaggacga

181 cacccagtgg ctgacttact gggtcg

//

**LOCUS ck48742** 158 bp mRNA linear

DEFINITION Colletotrichum kahawae strain CIFC Que2 40S ribosomal protein S28, mRNA partial sequence.

SOURCE Colletotrichum kahawae (anamorph: Colletotrichum kahawae)

FEATURES Location/Qualifiers

source 1..158

/organism="Colletotrichum kahawae"

/mol_type="mRNA"

/host="Coffea arabica"

/country="Kenya"

/anamorph="Colletotrichum kahawae"

CDS <1..101

/codon_start=3

/product="40S ribosomal protein S28"

/translation="QTRSIIRNVKGPVREDDILCLLESEREARRLR"

BASE COUNT 37 a 44 c 50 g 27 t

ORIGIN

1 accagacccg ttccatcatc cgtaacgtca agggacccgt ccgcgaggac gacattctct

61 gcttgctcga gtctgagcgt gaggcccgca gactgagata aaaaggcggc gacgatcggc

121 ggcggcgcgt gaatggacat tacgaggaac ggatcttg

//

**LOCUS ck36020** 140 bp mRNA linear 19-JAN-2016

DEFINITION Colletotrichum kahawae strain CIFC Que2 stf2-like protein, mRNA partial sequence.

SOURCE Colletotrichum kahawae (anamorph: Colletotrichum kahawae)

FEATURES Location/Qualifiers

source 1..140

/organism="Colletotrichum kahawae"

/mol_type="mRNA"

/host="Coffea arabica"

/country="Kenya"

/anamorph="Colletotrichum kahawae"

CDS <1..100

/codon_start=2

/product="stf2-like protein"

/translation="HGPNEEDGEDLTKTETSESGGSSYGDEKITKH"

BASE COUNT 33 a 43 c 41 g 23 t

ORIGIN

1 ccacggcccc aacgaggagg atggcgagga cctgaccaag acggagacct ccgagagcgg

61 cggttcctcc tatggtgacg agaagatcac caagcactaa tatcgggacg ctgtccctaa

121 tgtttcgtgc tgcagccctc

//

**LOCUS ck39066** 151 bp mRNA linear 19-JAN-2016

DEFINITION Colletotrichum kahawae strain CIFC Que2 hypothetical protein, mRNA partial sequence.

SOURCE Colletotrichum kahawae (anamorph: Colletotrichum kahawae)

FEATURES Location/Qualifiers

source 1..151

/organism="Colletotrichum kahawae"

/mol_type="mRNA"

/host="Coffea arabica"

/country="Kenya"

/anamorph="Colletotrichum kahawae"

CDS <1..>151

/codon_start=1

/product="hypothetical protein"

/translation="KGEWLKGLLPLDPAKAQEYNGGATVASPYQNPEDVLEWGWTPNV

YFFPYA"

BASE COUNT 36 a 37 c 47 g 31 t

ORIGIN

1 aagggtgaat ggttgaaggg tcttctgccc ctggatcccg caaaggcgca agaatataac

61 ggcggagcga cggttgccag cccgtatcag aacccggagg atgttttgga atggggctgg

121 acaccgaatg tctacttctt cccatacgca g

//

**LOCUS ck34620** 208 bp mRNA linear

DEFINITION Colletotrichum kahawae strain CIFC Que2 hypothetical protein, mRNA partial sequence.

SOURCE Colletotrichum kahawae (anamorph: Colletotrichum kahawae)

FEATURES Location/Qualifiers

source 1..208

/organism="Colletotrichum kahawae"

/mol_type="mRNA"

/host="Coffea arabica"

/country="Kenya"

/anamorph="Colletotrichum kahawae"

CDS <1..>208

/codon_start=1

/product="hypothetical protein"

/translation="PDFHFHYLTTTPEQVTRNYMEFIYKTYPLGSFDTRPLNFSDVSA

TLSIRRFLLDKIFQTFPQRKFILVG"

BASE COUNT 40 a 73 c 50 g 45 t

ORIGIN

1 cccgacttcc acttccatta cctgaccacc acgccggagc aggtgacgcg gaactacatg

61 gagttcatct acaagacgta ccccctgggc agcttcgata cccgtccgtt gaacttttcc

121 gacgtgagcg cgaccctgtc gatccgtcgg ttcctgctcg acaagatctt ccagacgttc

181 ccgcagcgca agttcatcct ggtcggcg

//

**LOCUS ck21238** 222 bp mRNA linear

DEFINITION Colletotrichum kahawae strain CIFC Que2 bifunctional catalase-peroxidase cat2, mRNA partial sequence.

SOURCE Colletotrichum kahawae (anamorph: Colletotrichum kahawae)

FEATURES Location/Qualifiers

source 1..222

/organism="Colletotrichum kahawae"

/mol_type="mRNA"

/host="Coffea arabica"

/country="Kenya"

/anamorph="Colletotrichum kahawae"

CDS <1..>222

/codon_start=2

/product="bifunctional catalase-peroxidase cat2"

/translation="SASTFRGSDKRGGANGARIRLAPQKDWKVNNPPQLKEVLQALEK

VQQKFNSSASGGKKVSLADLIVLGGVAGV"

BASE COUNT 42 a 60 c 67 g 53 t

ORIGIN

1 ttccgcatct accttccgtg gtagtgacaa gcgcggaggt gccaacggcg ctcgcatccg

61 cttggctccc cagaaggact ggaaggtcaa caaccctccc cagcttaagg aggtcctcca

121 ggcgcttgag aaggttcagc agaagttcaa cagctccgct tctggtggta agaaggtgtc

181 tttggccgac ttgatcgtcc ttggtggtgt tgctggtgtt ga

//

**LOCUS ck25805** 269 bp mRNA linear

DEFINITION Colletotrichum kahawae strain CIFC Que2 trihydroxynaphthalene reductase, mRNA partial sequence.

SOURCE Colletotrichum kahawae (anamorph: Colletotrichum kahawae)

FEATURES Location/Qualifiers

source 1..269

/organism="Colletotrichum kahawae"

/mol_type="mRNA"

/host="Coffea arabica"

/country="Kenya"

/anamorph="Colletotrichum kahawae"

CDS <1..207

/codon_start=1

/product="trihydroxynaphthalene reductase"

/translation="MYRDVCREYIPGGTELDDEGVDEYAAGWSPMHRVGLPIDIARVV CFLASQDGEWINGKVLGIDGAACM"

BASE COUNT 48 a 75 c 81 g 65 t

ORIGIN

1 atgtaccgtg atgtctgccg cgagtacatt cccggcggca ctgagctcga cgacgagggt

61 gtcgatgagt acgccgctgg ctggtccccc atgcaccgtg tcggtctccc catcgacatt

121 gcccgtgtcg tctgcttcct ggcttcccag gacggtgagt ggatcaacgg caaggtcctc

181 ggcatcgacg gtgccgcctg catgtaaatt gctacctaat ataatcggag ttacgggtta

241 ctggggctgg tattagtaga ttggatcac

//
