# Supplementary material for: Comparative Validation of Conventional and RNA-Seq Data-Derived Reference Genes for qPCR Expression Studies of Colletotrichum kahawae
Source: PLoS One. 2016 Mar 7;11(3):e0150651. doi: 10.1371/journal.pone.0150651 (PMC4780792; doi:10.1371/journal.pone.0150651)
Supplement: S2 File — (DOC) [file pone.0150651.s005.doc]

**S2 File. - Primer specificity test through dissociation curve analysis collected from iQ5 (Bio-rad) using several samples of *C. kahawae* and *C. arabica – C. kahawae.***

| *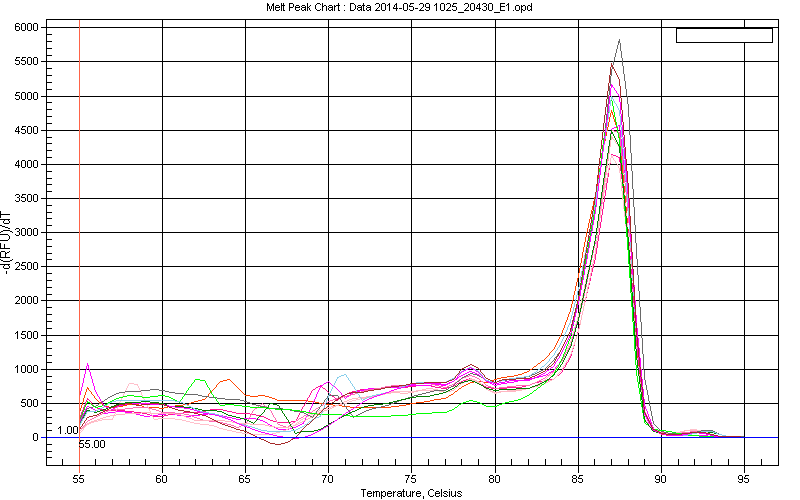ck20430* | *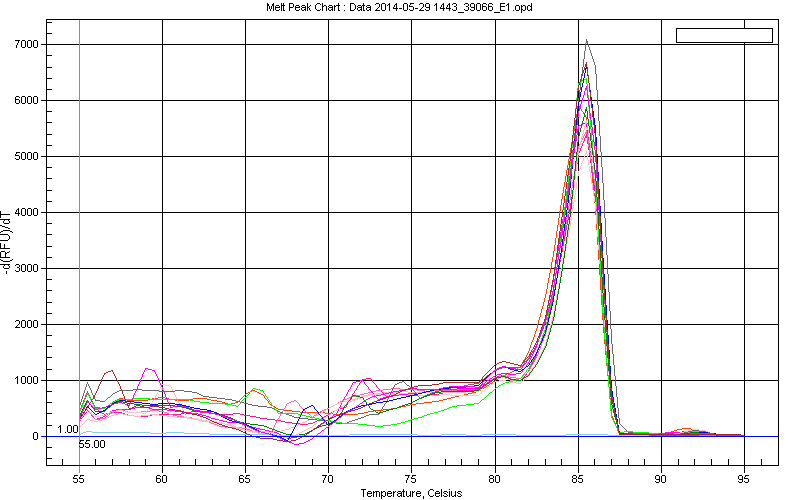ck39066 – DNA* |
| --- | --- |
| 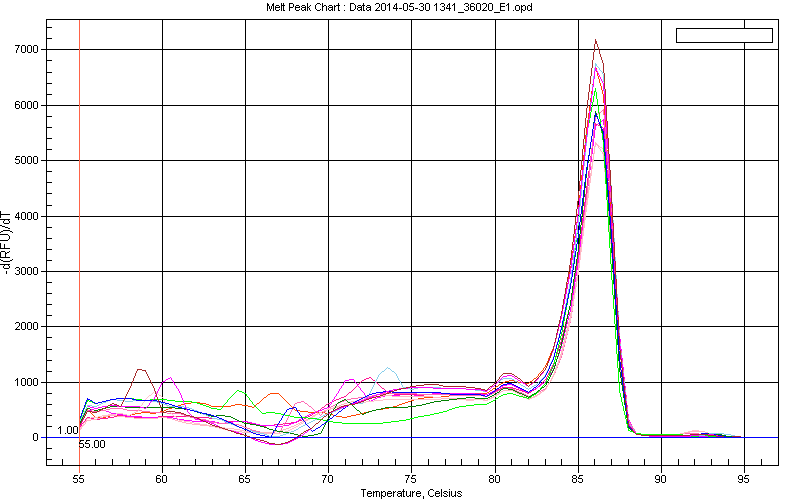ck*36020* | *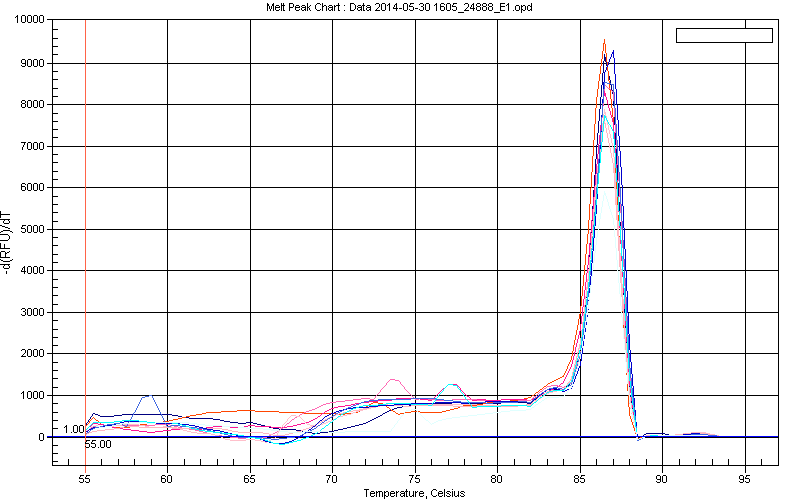ck28444* |
| *PP1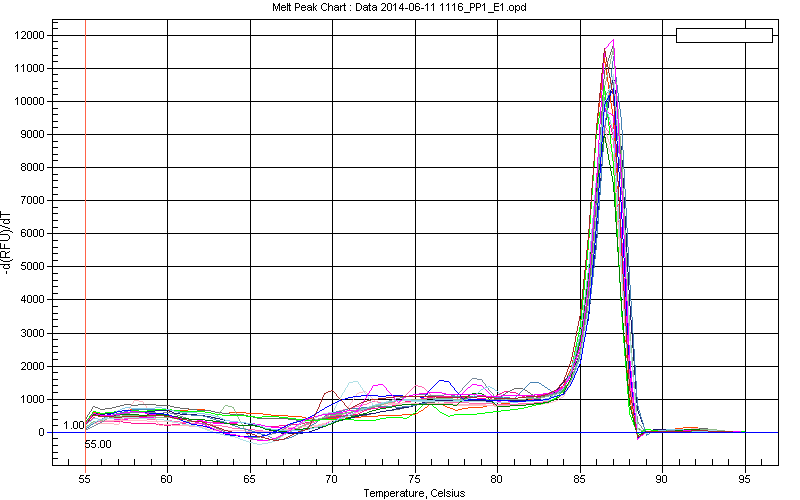* | *Act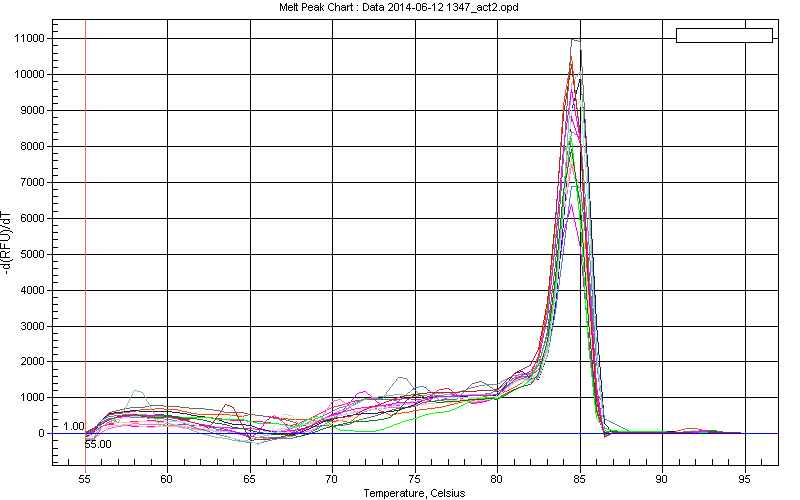* |
| *Cyp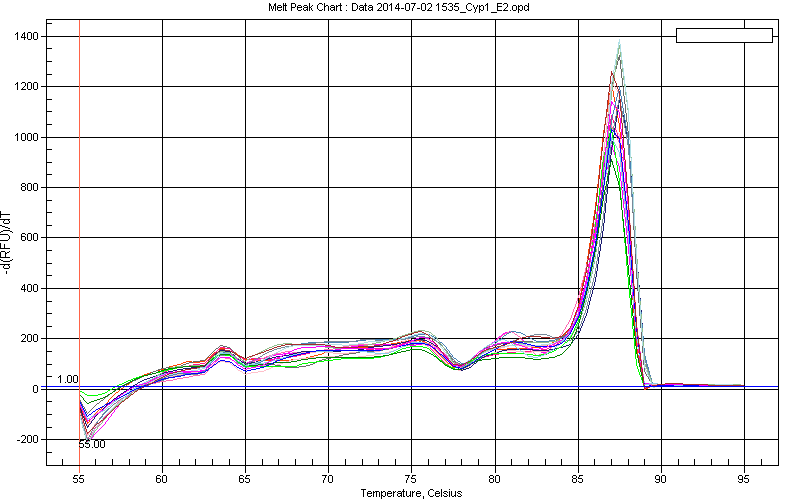* | *ck 48742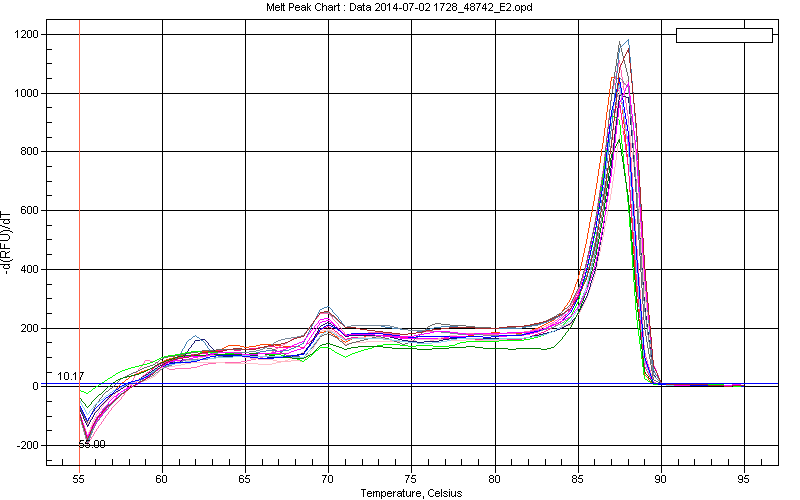* |
| *ck34620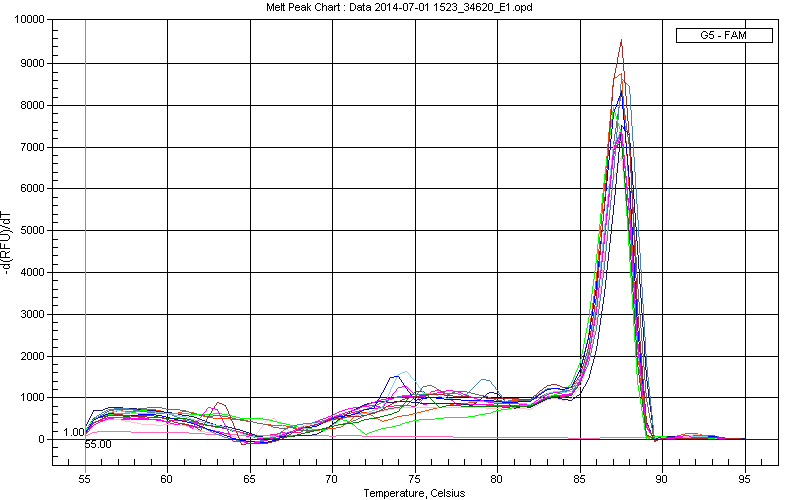* | *thr1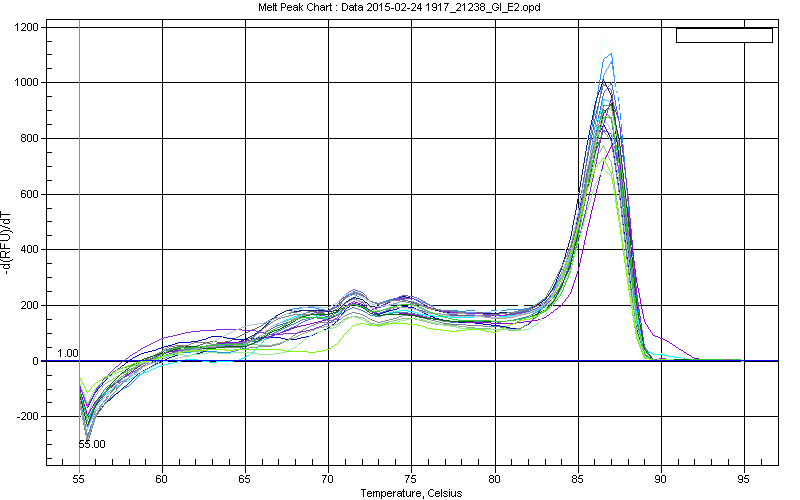* |
| *cat2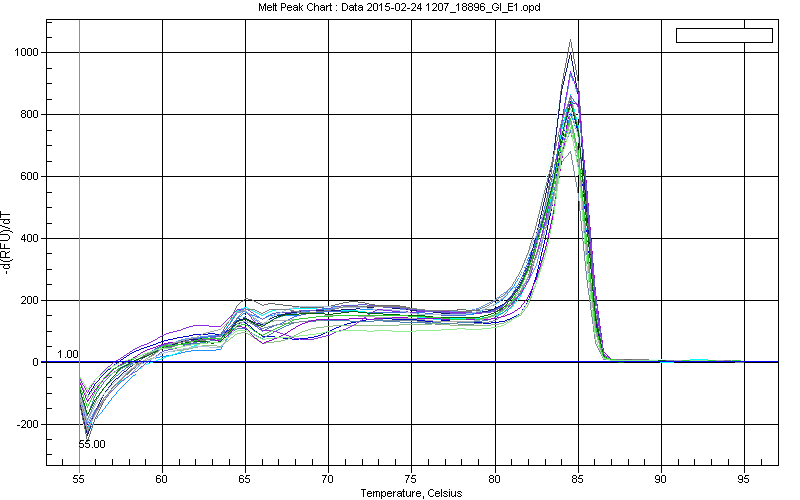* |  |
